# Goal setting is insufficiently recognised as an essential part of shared decision-making in the complex care of older patients: a framework analysis

**DOI:** 10.1186/s12875-019-0966-z

**Published:** 2019-06-06

**Authors:** Neeltje Vermunt, Glyn Elwyn, Gert Westert, Mirjam Harmsen, Marcel Olde Rikkert, Marjan Meinders

**Affiliations:** 1 Radboud University Medical Center, Radboud Institute for Health Sciences, Scientific Institute for Quality of Healthcare (IQ Healthcare), PO Box 9101, 6500 HB Nijmegen, The Netherlands; 2 The Dutch Council for Health and Society, (Raad voor Volksgezondheid en Samenleving, RVS), The Hague, the Netherlands; 3 The Dartmouth Institute for Health Policy & Clinical Practice, Level 5 Williamson Translational Research Building, One Medical Center Drive, Lebanon, NH 03756 USA; 4 Cochrane Institute for Primary Care and Public Health, Cardiff University, Cardiff, UK; 5 Radboud university Medical Center, Radboudumc Alzheimer Center, Donders Institute for Brain, Cognition and Behavior, PO Box 9101, 6500 HB Nijmegen, The Netherlands; 6 Department of Geriatrics, Radboud University Medical Center, PO Box 9101, 6500 HB Nijmegen, The Netherlands

**Keywords:** Shared decision-making, Goal setting, Goals, Multimorbidity, Older patients, General clinicians, Clinical geriatricians

## Abstract

**Background:**

Multimorbidity poses a challenge for decision-making processes and requires that more attention is paid to patient goals, preferences and needs; however, goal setting is not yet widely recognised as a core aspect of the shared decision-making (SDM) approach. This study aims to analyse clinician perceptions of the concept of goal setting within the context of SDM with older patients with multimorbidity.

**Methods:**

Semi-structured interviews with general practitioners (GPs) and clinical geriatricians (CGs) were analysed using a framework analysis. The integrative model of SDM was used to develop a categorisation matrix, including goal setting as an additional component.

**Results:**

Sixteen of the 33 clinicians mentioned explicit *Goal setting* as an integrated component of their definition of SDM, which was comparable to the number of clinicians who listed *Patient values and preferences* (*n* = 16), *Doctor knowledge and recommendations* (*n* = 19) and *Make or explicitly defer a decision* (*n* = 19), elements which are commonly considered to be important aspects of SDM. The other 17 clinicians (6 CGs and 11 GPs) did not mention *Goal setting* as an explicit component of SDM. Our analysis revealed two potential reasons for this observation. Besides the use of other terminology, part of clinicians viewed collaborative goal setting and SDM as separate but related processes.

**Conclusions:**

Our study on clinician perspectives highlighted goal setting as component of a SDM approach and could therefore be considered supportive of recent theoretical insights that SDM models that lack an explicit goal-setting component appear to be deficient and overlook an important aspect of engaging patients in decision-making, particularly for patients with complex multimorbidities. We therefore call for the further development of a comprehensive SDM approach for older patients with multimorbidity to include explicit and unequivocal goal setting elements to sufficiently meet the expectations and needs of clinicians and their patients.

**Electronic supplementary material:**

The online version of this article (10.1186/s12875-019-0966-z) contains supplementary material, which is available to authorized users.

## Background

Goal setting has emerged as a critical aspect of the care of patients with two or more long-term conditions, yet little attention had been paid to patient goals in the approach known as shared decision-making (SDM). Is this an oversight or was it deliberate? Where there is multimorbidity, defined as the co-existence of two or more chronic diseases or conditions, clinical priorities can compete with one another, and patients can bring additional perspectives and priorities into consideration [1–10]. To address these challenges, many have advocated paying attention to patient goals, preferences and needs [11–14]. Tinetti et al. recommended a healthcare shift from a disease orientation to a patient goal orientation [7], which requires that patient priorities and goals are ascertained and the problems impeding these goals are identified. Explicit goal setting might improve the process and outcomes of decision-making in complex cases. In the context of ageing populations, a goal-oriented approach to healthcare could be beneficial at many levels [7, 8, 15–17].

Shared decision-making (SDM) would seem highly compatible with taking a goal-oriented approach to care, yet the role of goals and goal setting were not explicitly described in these models [18–21]. Based on their 2006 analysis of five prominently cited SDM models, Makoul and Clayman [18] identified the most frequently invoked elements and qualities of these approaches and presented an integrative model for SDM. This integrative model is restricted to the essential elements, as presented in Table 1, and is intended to encompass different clinical contexts, types of decisions and levels of involvement.

**Table 1:** Essential elements, ideal elements and general qualities of SDM

Essential elements	Ideal elements	General qualities
Define/explain problem	Unbiased information	Deliberation/negotiation
Present options	Define roles	Flexibility
Discuss pros/cons	Present evidence	Information exchange
Patient values/preferences	Mutual agreement	Involves at least two people
Discuss patient ability/self-efficacy		Middle ground
Doctor knowledge/recommendations		Mutual respect
Check/clarify understanding		Partnership
Make or explicitly defer decision		Patient education
Arrange follow-up		Patient participation
		Process/stages

*Note*: Table 1 provides an overview of essential elements, ideal elements and general qualities of SDM, based on the research of Makoul and Clayman [18]. Their integrative model of SDM is restricted to the essential elements because it was intended to encompass different clinical contexts, types of decisions and levels of involvement. The ideal elements may enhance the SDM process but are more applicable to some encounters than others, and not necessary for SDM to take place. The general qualities provide an overall sense of SDM; however, these are not specific to SDM

Bodenheimer and Handley defined the term collaborative goal setting (CGS) as ‘a process by which healthcare professionals and patients agree on a health-related goal’ [22]. This enables the acknowledgment of health-related goals and goal setting in the context of behaviour change and action planning for chronic conditions in primary care settings, without necessarily relating them to SDM. CGS has been evaluated in various rehabilitation settings [23–26]. In a 2014 Commonwealth Fund Survey of adults aged 65 or older with at least one chronic condition, the number of patients who stated that they shared their health goals with a healthcare professional ranged from 23% (Sweden) to 59% (UK), with 9 of the 11 countries having rates below 50% [27]. Furthermore, relatively few studies have explored the processes used by clinicians to set goals in the presence of complex multimorbidity, resulting in little evidence to support the best practices for goal setting with these patients [28]. In a recent systematic review, we concluded that CGS is often a component of complex multifactorial interventions [29]; however, the use of CGS in the daily practice of caring for older patients with multimorbidity is still evolving.

Increasing numbers of researchers have noted the absence of goal setting in SDM models [27–35], and changes are being introduced to try to address this deficit. In 2012, Elwyn et al. developed a SDM model consisting of a Choice Talk, an Option Talk and a Decision Talk, which were later updated to Team Talk, Option Talk and Decision Talk [21, 36]. In 2017, this three-talk model was revised to incorporate goal setting as an element of the first step of the SDM process, the Team Talk [35]. Van de Pol et al. [31] proposed a SDM model particularly suited for use with older patients with multimorbidity that built on this three-step model by adding a fourth term, Goal Talk. The conceptual link between CGS and SDM is not self-evident however; for example, Rose et al. [37] conducted a systematic review on SDM *within* CGS in rehabilitation settings.

It is clear that a coherent description of how best to accomplish SDM with older patients who have complex multimorbidities has not yet been fully established. Although absent from early models of SDM, integrating goal setting and goal attainment in a SDM approach might improve the accomplishment of care plans for patients who need to juggle the burden of illness against the burden of treatments; however, it is currently unclear how goal setting fits into the concept of SDM. Furthermore, the potential adaptations of an approach must not lead to an unnecessary increase in the complexity of its use in daily practice. The inclusion of goal setting would imply that it is a necessary and maybe fundamental aspect of SDM that would be expected to improve the suitability of SDM for use with complex cases, a benefit that will likely outweigh the potential risks of making such a change.

General practitioners (GPs) and clinical geriatricians (CGs) may be able to use their experience of daily care and decision-making with older patients with multimorbidity to better elucidate the relationship between SDM and goal setting. The aim of the present study was therefore to examine whether clinicians view goal setting as a component of SDM and, if so, whether the care of patients might be facilitated by integrating explicit goal setting into a SDM approach. For this purpose, we conducted and analysed interviews with GPs and CGs using a framework approach [38, 40].

## Methods

A qualitative study was conducted based on semi-structured interviews with expert CGs and GPs. The interviews were analysed in two phases, beginning with a thematic analysis [39] of all interview topics followed by an analysis of certain themes derived specifically for the purpose of this study using a framework approach [38, 40]. For the framework approach, the integrative model of SDM developed by Makoul and Clayman [18] with the additional component of goal setting was used as the categorisation matrix. The Consolidated Criteria for Reporting Qualitative Research (COREQ) [41] and the Guidelines for the authors and reviewers of qualitative studies [42] were used for the design, performance and reporting of these analyses. The COREQ criteria are reported in terms of our research in Additional file 1. Atlas-ti 7.1.15 software was used in the data coding and analysis. This paper reports on the framework analysis of the interviews.

### Sampling

A purposive and snowball method was used to recruit potential participants, all of whom were professional experts in geriatric care in hospital and community settings in the Netherlands [43]. These included GPs specialised and experienced in geriatric care, as well as experienced CGs working in an academic or non-academic teaching hospitals, performing research, teaching, developing or implementing specific innovations in the care of older patients. The sampling was targeted to ensure that a comparable number of GPs and CGs would be included. To obtain diverse perspectives, different types of practice and practice location (rural or urban) were represented in the GP sample, while CGs were recruited from different types of hospitals. For both the CGs and GPs, as many Dutch regions as possible were represented. The sampling of potential participants was initiated by interviewing a GP and a CG, both of whom were familiar to the interviewer. At the end of the interview, the interviewees were asked to suggest the names of potential interview participants, including both GPs and CGs. Recruiting potential participants while also taking into account geographical spread appeared to be easier for the CGs than the GPs, probably because the CGs were hospital-based with a regional focus. GPs were also recruited at a meeting of GPs specialised in geriatric care, which allowed for a wider regional spread of potential participants and avoided regional clustering. The potential participants were approached by email.

### Data collection

An interview guide was drafted based on two viewpoints about goal-oriented healthcare for older patients with chronic multimorbidity [7, 14]. The main topics were SDM, CGS and effective collaborative action. *Effective collaborative action* was defined as the clinicians and patient jointly deciding on and performing diagnostic and treatment steps in line with collaborative goals, which were established by the patient and their clinicians or other involved caretakers. Definitions were not provided to the interviewees. The main topics and subtopics are presented in Additional file 2.

In the introduction of the interviews, the clinicians were asked to use the context of their regular care for community-dwelling older patients (age > 75 years) with a chronic disease or multimorbidity without any further specifications. Specific questions could be altered to obtain a better understanding of certain (sub)topics. All interviews were conducted by the same interviewer (NV) and lasted approximately 60 min. Two pilot interviews were conducted with a CG and a GP. The main topics and subtopics were not changed based on the pilot interview nor during the conducting of the interviews. Five interviews were held face to face, but the others were held by telephone, as the clinicians’ busy schedules and varying locations required flexibility. The face-to-face interviews were held at the interviewee’s office. All interviews were audio-recorded and transcribed. Field notes were kept and analytical memos were drafted during data collection and analysis. The sample size was guided by theoretical saturation [44]. After no new data were determined to have arisen, two further interviews were conducted as a confirmation.

### Analysis

In the first phase, a thematic analysis was performed [39] for all interview topics, after which a framework approach was taken for the purposes of this study [38, 40]. Thematic analyses focus on identifying, analysing and reporting patterns (themes) within qualitative data and on the interpretation of aspects of the research topics. During the interviewing phase, NV and MH conducted preliminary analyses by reflecting and discussing the interviews and field notes. The interview guide was adapted to reflect their emerging insights. Open coding was applied to all topics of the first five transcripts, which was independently performed by two data coders (NV and MH). The data were conceptually interpreted and labelled accordingly, resulting in initial codes which were then compared, discussed, grouped and categorised to determine a working coding tree. The remaining interviews were coded by one researcher (MH) and checked by the other (NV). In weekly meetings, the coding of the transcripts was compared, discussed and agreed upon by the researchers (NV and MH), which included the creation of additional codes and the further refinement of the analysis taking reflexivity into account. The findings were discussed and further adjusted after review by the broader research team consisting of all co-authors. Amongst others, this thematic analysis of the interview data resulted in the themes ‘SDM concept’ and ‘Links between the concepts of SDM and CGS’.

In the second phase of the analysis, these themes were further analysed using a framework approach. For this purpose, a SDM categorisation matrix was developed based on the essential, ideal and general elements of the integrative model of SDM developed by Makoul and Clayman [18], shown in Table 1. A new category, goals/goal setting, was added to this SDM categorisation matrix. The data were charted in this categorisation matrix and the findings were interpreted. Goals/goals setting had to be mentioned specifically before they were categorised as being ‘mentioned’.

The findings were discussed in regular meetings between NV and MM and further adjusted after review by the broader research team consisting of all co-authors. Illustrative quotations were selected to highlight the findings and translated from Dutch to English by a professional translator.

### Ethical considerations

At the start of the interview, all interviewees were informed about the study, the method of data handling and the preservation of anonymity. Permission to record audio during the interviews was obtained from all participants. In addition to their earlier consent to be interviewed, documented in the emails, all interviewees were formally asked for their consent to participate, which was audio recorded and documented by the interviewer. To preserve anonymity and confidentiality, names were replaced with codes indicating whether the participant was a GP or CG and the sequential number of the interview. Any identifying information was removed following the verbatim transcription of the recordings.

## Results

### Interview and participant characteristics

The response rates of the CGs and GPs were 86 and 54%, respectively, resulting in a final sample of 33 clinicians (18 CGs and 15 GPs). The first author (NV), a former GP, conducted the interviews between November 2012 and April 2013. Five interviews were conducted face to face, while the remaining 28 were held over the telephone. All interviews lasted approximately 60 min. The mean age of the GPs was 51 (*n* = 15), while for the CGs (*n* = 18) it was 48; 60% of GPs and 50% of CGs were female. On average, the GPs had 16 years of professional experience and CGs had 10 years. Additional participant characteristics are presented in Table 2.

**Table 2:** Basic characteristics of the participants

Characteristics	General practitioner *(n = 15)*	Clinical geriatrician *(n = 18)*
Age, *mean (SD)* (years)	51 (6.6)	48 (8.6)
Gender, *n* (% women)	9 (60)	9 (50)
Practice type, *n (*%)		N/A
Single	1 (7)	
Joint partnership	2 (13)	
Group/health centre	12 (80)	
Practice location, *n* (%)		N/A
Rural area	3 (20)	
Urbanised rural area	5 (33)	
Urban area	7 (47)	
Physician assistant in geriatric care^a^, *n* (% yes)	12 (80)	N/A
Type of hospital, *n* (%)	N/A	
Academic centre		3 (17)
Community hospital		9 (50)
Mental care facility		2 (11)
Non-academic teaching hospital		4 (22)
Researcher, *n* (% yes)	5 (33)	9 (50)
Supervisor, *n* (% yes)	3 (20)	11 (61)
GP specialised in geriatric care, *n* (% yes)	9 (60)	N/A
Years of professional experience, *median (range)*	16 (3–34)	10 (3–22)

*N/A* not applicable, *SD* standard deviation, *GP* general practitioner

^a^in GP practice

### Main elements of SDM according to CGs and GPs

In 2006, Makoul and Clayman [18] identified the most frequently invoked elements and qualities of SDM and developed an integrative model of this approach, which is presented in Table 1. As already has been argued, Makoul and Clayman’s integrative model is intended to encompass different clinical contexts, types of decisions and levels of involvement. To investigate whether clinicians mention goal setting as an essential component of SDM, we added the component of *Goal setting* to the integrative model to constitute a categorisation matrix.

Table 3 provides information on which elements of the integrative model and the additional component of goal setting were mentioned by each clinician. Although not included in the original integrative SDM model, *Goal setting* was explicitly mentioned by half of the clinicians interviewed (*n* = 16). Established important components of SDM, which were also listed were: *Patient values/preferences* (*n* = 16), *Doctor knowledge/recommendations* (*n* = 19) and *Make or explicitly defer decision* (*n* = 19).

**Table 3:** Categorisation matrix for SDM with older patients with multimorbidity

		GP_01	CG_02	CG_03	GP_04	CG_05	GP_06	CG_07	CG_08	GP_09	GP_10	CG_11	CG_12	GP_13	CG_14	GP_15	CG_16	CG_17	CG_18	CG_19	GP_20	GP_21	CG_22	GP_23	GP_24	CG_25	GP_26	GP_27	GP_28	CG_29	CG_30	GP_31	CG_32	CG_33
Essential elements																																		
Define/explain problem					x	x							x							x				x			x		x		x		x	x
Present options				x			x		x	x		x	x		x			x											x		x		x	
Discuss pros/cons^a^						x		x	x	x		x						x									x		x					
Patient values/preferences		x		x	x	x		x	x	x	x			x	x		x					x	x				x		x	x	x			
Discuss patient ability/self-efficacy					x																													
Doctor knowledge^b^		x		x	x	x	x	x	x		x		x	x	x		x						x				x		x	x	x		x	x
Check/clarify understanding										x	x			x																				
Make or explicitly defer decision							x			x	x	x	x	x		x		x	x	x		x	x	x	x	x	x		x		x		x	
Arrange follow-up						x																				x								
Ideal elements																																		
Unbiased information				x		x																	x											
Define roles ^c^																																		
Present evidence											x																							
Mutual agreement							x			x	x					x		x		x	x	x						x						
General qualities																																		
Deliberation/negotiation					x			x					x	x	x	x					x			x			x		x	x				
Flexibility ^d^					x	x						x	x						x															
Information exchange								x							x																			
Involves at least two people																																		
Middle ground																																		
Mutual respect														x																				
Partnership			x		x														x	x	x			x		x				x			x	x
Patient education																																		
Patient participation			x							x	x	x		x			x	x				x	x		x					x				
Process/stages		x																																
Goals and/or goal-setting			x	x	x	x		x				x	x				x		x	x	x					x		x		x		x	x	

*Note*: This table shows the categorisation matrix and results per clinician. The integrative model of shared decision-making of Makoul and Clayman [18] completed with the additional element goals and/or goal-setting was used as categorisation matrix. At the horizontal axis the interviewees are shown, with GP referring to a general practitioner and CG to clinical geriatrician. The vertical axis shows the essential elements, ideal elements, general qualities and the additional element goals and/or goal-setting. Essential elements are elements being conditional for the process of SDM. Ideal elements may enhance the experience of SDM, but relevance, necessity and applicability are dependent on the type of encounter. General qualities are useful in providing an overall sense of SDM, but are not reducible to specific behavior

^a^/benefits/risks/costs

^b^ and recommendations

^c^and desire for involvement

^d^/individualised approach/cyclical

#### Goal setting as a component of SDM

The clinicians who considered goals and/or goal setting to be a component of SDM emphasised several aspects of these categories in their descriptions. Goals and the goal setting process were described as providing inputs for the decision-making process, as illustrated by the following quotation:

*“Once you have gained, let’s say, insight into the patient’s goals and the doctor’s possibilities, then you can reach a decision. (...) In the process of exchanging information, the patient is already telling you about how they lead their life, about their goals, wishes and desires, etc. And (...) what they want to do with those”.* Clinical geriatrician 7 (CG_07)

Furthermore, goals and goal setting can be seen as core components of SDM, reflecting the essence of a SDM approach, as explained by one of the clinicians:

*“If you opt for shared decision-making, I think you should start by looking at what the actual goal is: ‘What is the patient’s goal?’ (...) I believe that is what the entire process of shared decision-making is about”.* Clinical geriatrician 11 (CG_11)

Similarly, one of the clinicians stated:

*“I believe that you cannot make common decisions if you do not have joint goals”.* Clinical geriatrician 16 (CG_16)

In this sense, goals and goal setting reflect a person-centred attitude. Person-centred care is defined as healthcare that is ‘respectful and responsive to individual patient preferences, needs and values, and ensures that patient values guide all clinical decisions’ [45]. SDM can be seen as fundamental to patient-centred care [46], and both explicitly involve the patient in their care [47, 48].

Furthermore, the integration of the CGS and SDM processes was emphasised in our analysis. According to some of the clinicians, CGS and SDM can be viewed as a single integrated process and cannot be separated, since decision-making also involves goal setting. They stated that the patient and clinician should jointly determine which goals are relevant and which steps should be taken in pursuing those goals:

*“When you ask someone: ‘What do you think about your life and what do you find most important?’, and it turns out that ‘continue to live independently’ is the most important, then it makes sense that the patient is involved in thinking about the ‘smaller’ goal that is linked to this (...) and that they also give the green light to this... for example, by asking ‘Are you okay with this? And if you’re not okay with THIS, how would you feel about THAT?’. This is all interwoven and cannot be seen separately”.* General practitioner 20 (GP_20)

This general practitioner seems to refer to various types of goals. Independence can be seen as a value and ‘continuing to live independently’ is a goal incorporating this value. This goal setting process could be interpreted as a process of setting of several related goals within a decision-making process. In the view of some clinicians, goals can actively steer the options presented to a patient, as illustrated by the following quotation:

*“If someone says: ‘I want to continue living independently as long as possible’, well, then perhaps those treatment options must be chosen that enable the patient to do this. Very invasive options, meaning for example that a patient has to travel back and forth constantly, are then excluded. (...) It means you choose the more pragmatic option. (...) Yes, I do believe that such goals give direction to the options. So if you know those goals in advance and you know what the patient wants, it becomes easier to give better advice about what the best option is”.* Clinical geriatrician 3 (CG_03)

This value-driven goal of ‘continue to live independently as long as possible’ could be used to steer the decision-making process and enable the comparison of certain options.

#### Perceptions of clinicians not mentioning goal setting as a component of SDM

Seventeen clinicians did not explicitly mention goal setting as a component of a SDM approach. Of the clinicians who did not explicitly mention goal setting as part of SDM, six were CGs and 11 were GPs. This means that, in contrast to the CGs, the majority of GPs did not explicitly mention goal setting as a SDM component. Our analysis revealed some indication that the lack of an explicit mention of goals when attempting to undertake a SDM process does not automatically mean that there is a lack of awareness regarding goals. First, some clinicians who did not mention goals explicitly focused on other aspects of patient involvement, using terms such as ‘agreement’ or ‘decision maker’ in their description of the concept of SDM. They mentioned that aligning decisions with a patient’s preferences is essential:

*“In any case, to align our thoughts. (...) to make my plan clear to the patient or ask what they still want. (...) and check (...) whether the patient understands what I mean, as a GP, by a certain proposal”.* General practitioner 13 (GP_13)

We can interpret these aspects of patient involvement as elements of a person-centred attitude. In this sense, the attitudes of the clinicians who did or did not mention goal setting are not necessarily different. As was also concluded by Knight et al., the concepts of values, goals, and preferences are often used interchangeably [49], which could explain why some clinicians did not mention goals or goal setting.

Some clinicians did not mention goal setting when defining SDM but described CGS and SDM as separate but related processes. Some of them see CGS and goals as fundamental and SDM as a related, though more concrete, process of decision-making:

*“[SDM and CGS] are two different stories, of course. Yes, because when you set a goal, you ask ‘What is important to you?’ (...) ‘When you think of the next couple of years, what is it that you want or don’t want?’ And decision-making simply means that you involve the patient in the choices that you make. (...) And (...) that you provide the information the patient needs to oversee things and (...) that you try to reach a satisfactory result together. (...) But that is just a little bit different. (...) Yes, I think decision-making is bigger than that. (...) And (...) to reach a decision together, that also serves that joint goal, now doesn’t it”.* General practitioner 17 (GP_17)

Although viewed as separate processes, from this perspective CGS remains a key input for a SDM process.

## Discussion

Goal setting is not yet widely recognised as a core component of SDM. Our interviews with experienced GPs and CGs highlight the need for SDM approaches that explicitly and unequivocally include the task of goal setting. This would be consistent with recent theoretical insights that patient goals and the process of goal setting can be regarded as a fundamental part of a SDM process, especially when patients have multiple long-term conditions which require trade-offs between treatment options.

This study has some methodological strengths. First, we worked with an interviewer who is trained as a GP, which may have encouraged the participants to speak frankly and directly from their own professional perspectives. The second coder of the first phase was experienced in interview analysis but has no medical background. The second data analyst during the second phase (MM) is an expert on SDM, but has no background in practicing medicine. These distinct professional backgrounds helped us avoid a ‘medical’ bias in our data interpretation. Second, this topic can be considered as early research into this topic. At the time of our interviews, goal setting in complex decision-making was only just evolving, which is why we used a purposive sampling and a snowball extension method to recruit professional experts. In the Netherlands, both GPs and CGs provide medical care to older people living at home, but they do so in different settings, namely in the community and hospitals, respectively. At this stage of theory development, we considered their work to be complementary, allowing both to contribute to the saturation of data collection on current medical thinking on these themes. The basic characteristics of the participants showed considerable variability in line with the Dutch context (e.g., in terms of practice type). Third, we chose a framework analysis method, which is well-suited for an analysis that departs from a theoretical position. In our analysis we used a SDM categorisation matrix based on the integrative SDM model of Makoul and Clayman.

This study also has some limitations. We aimed to focus the interviews on the care of a population with complex healthcare demands, which was introduced to the interviewees as a population of people over 75 years of age with multimorbidity. Ideally, in the context of condition and functioning, we would have been able to specify this potential complexity further by also including factors such as disease severity and disability [50]; however, we assumed that this might complicate the interviews too much if it was taken too literally. Patients and/or caregivers were not interviewed as part of this research, but should be included in future research on this topic because it centres on patient participation and the patient orientation of care. Furthermore, we found a difference between the proportion of GPs and CGs who mentioned goal setting. We have no reason to believe that GPs are less patient-oriented than CGs; in a qualitative focus group study of Dutch GPs, Luijks et al. [4] reported that GPs agreed to involve patient perspectives and preferences in the decision-making process. Further research is necessary to determine whether this indication of possible differences in perceptions between GPs and CGs can be confirmed and if so, what causal factors could be relevant in explaining these differences. In addition, we used spontaneous descriptions of the SDM context in our analysis of whether the clinician mentioned goal setting. Furthermore, although the interview guide started with the topic of SDM, all topics, including CGS, were mentioned in the introduction of the interviews, which could have primed the interviewees. Finally, although our findings contribute to the current academic debate on goal setting and SDM, the time span between interviewing and publishing the results could also be considered a limitation. Findings might be somewhat different nowadays, as the SDM field has evolved, including the consideration of goal setting as element of SDM, since the original study was conducted.

Although there is still a relative lack of tools and evidence regarding the effects of goal setting with older patients with chronic disease or multimorbidity [29], we would like to appeal for the integration of explicit goal setting into SDM approaches for older patients with multimorbidity for three reasons: 1. The potential relevance of the inclusion of goal setting as essential component; 2. the need for consistent terminology and 3. the need for the further development of practical goal setting within SDM. These reasons are explained in more detail below.

First, our findings are in line with recently published theoretical insights on the relevance of goal setting in a SDM approach [27–35]. Sixteen of the 33 clinicians interviewed mentioned *Goal setting* as a component of their definition of a SDM approach. The frequency of the inclusion of *Goal setting* as a SDM component is comparable with the mention of the other commonly accepted major elements, namely *Patient values/preferences* (*n* = 16), *Doctor knowledge/recommendations* (*n* = 19) and *Make or explicitly defer decision* (*n* = 19). Furthermore, the interpretation of the descriptions of goal setting revealed that the interviewees who mentioned goal setting considered it to be an important component.

A need for consistency in terminology is a second reason why we believe goal setting should be incorporated in SDM approaches. As our findings indicated, clinicians seem to use different terms in their definition of SDM, which is consistent with other research showing that the concepts of values, goals, and preferences are often used interchangeably [49]. Clearly defining these concepts and their relevance within SDM will contribute to the further development of consistent theoretical models and practical approaches. In our view, special attention should be given to defining the types of goals in the goal setting process, as our results indicated that clinicians may set and use varying types of goals.

The third reason for our plea is the need for the further development of practical approaches for goal setting within SDM. Clinicians are increasingly interested in goal-oriented care, especially for patients with multimorbidity [1, 7, 8, 11, 14, 51, 52]. A goal-oriented approach in decision-making can be helpful in personalising care to accommodate patient goals, preferences and resources [13, 14]. As argued in the introduction, CGS has only recently been incorporated into the daily practice of caring for older patients with multimorbidity, and incorporating patient values, preferences and circumstances is a difficult step in the decision-making process [53]. Health-related goals arise not only from health but also from other dimensions such as social context or wellbeing [1, 13, 14, 51]. Clinicians may struggle to help people prioritise their values, define treatment goals and frame preferences in ways that are clinically relevant and personally meaningful (aligned with one’s values) when faced with multiple diagnostic and treatment options [2, 54]. Based on our findings, we therefore want to make a plea for the integration of goal setting into the SDM model for use with older patients with multimorbidity as a next step to constitute a solid theoretical base for the further development of practical tools for facing these challenges. The perspectives of the interviewed clinicians reported here suggest that the explicit inclusion of goal setting *as part of* a SDM approach seems to be most promising to synchronise the concepts and approaches in caring for this category of patients.

Our research has several implications. Our findings seem to indicate that clinicians who want to practice SDM in the complex group of older patients with multimorbidity might be facilitated by the explicit integration of goal setting into this approach, although further research into the perceptions of patients regarding this topic is necessary. Furthermore, the potential differences in the perceptions of GPs and CGs require further elucidation. Finally, for practical tool development, the potential variation in the types of goals is an important topic for further research. Integrating explicit goal setting into a SDM approach would probably increase the use of goal setting in practice, and may lead to further tool development. These goals could enable the development of unambiguous terminology for the inclusion of patient perspectives in SDM. It might also improve inter-professional communication and collaboration by offering the possibility of exchanging explicit goals. In our view, the awareness and integration of goal setting in SDM could be beneficial for all patient categories, although the benefits may vary. We expect that the more complex the decision-making process becomes, the more beneficial explicit goal setting will be.

## Conclusions

Our study on clinician perspectives highlighted a lack of explicit goal setting as component of a SDM approach. We conclude that a comprehensive SDM model for use with older patients with multimorbidity could be developed further by including an explicit goal setting process, which is regarded by clinicians as the key factor in aligning diagnostic and therapeutic options with patient preferences and values.

## Additional files

Additional file 1: **Table S1.** Consolidated Criteria for Reporting Qualitative Studies (COREQ). Consolidated Criteria for Reporting Qualitative Studies (COREQ): A 32-item checklist [41]. (DOCX 30 kb)

Additional file 2: **Table S2.** Main topics and subtopics of the semi-structured interview guide. (DOCX 21 kb)

## Data Availability

The anonymised interview transcripts (in Dutch) supporting the conclusions of this article are available upon request by contacting the first author (NV).

## Abbreviations

CG: Clinical geriatrician
CGS: Collaborative goal setting
GP: General practitioner
SDM: Shared decision-making
