# Supplementary material for: Goal setting is insufficiently recognised as an essential part of shared decision-making in the complex care of older patients: a framework analysis
Source: BMC Fam Pract. 2019 Jun 6;20:76. doi: 10.1186/s12875-019-0966-z (PMC6555756; doi:10.1186/s12875-019-0966-z)
Supplement: Supplementary file 1 — Table S1. Consolidated Criteria for Reporting Qualitative Studies (COREQ). Consolidated Criteria for Reporting Qualitative Studies (COREQ): A 32-item checklist [41]. (DOCX 30 kb) [file 12875_2019_966_MOESM1_ESM.docx]

| **Table S1.** Consolidated Criteria for Reporting Qualitative Studies (COREQ) | | |
| --- | --- | --- |
| No | Item | Answer |
| Domain 1: Research team and reflexivity |  |  |
| Personal characteristics |  |  |
| 1. | Interviewer/facilitator | First author |
| 2. | Credentials | MD, MSc |
| 3. | Occupation | Senior advisor at an advisory council on public health and healthcare (public service) and PhD student. |
| 4. | Gender | Female |
| 5. | Experience and training | Economist and former general practitioner. PhD student. Courses on Atlas-ti and qualitative research. |
| Relationship with participants |  |  |
| 6. | Relationship established | The sampling of potential participants was initiated by interviewing a GP and CG, who were both acquaintances of the interviewer; the other participants were not. |
| 7. | Participant knowledge of the interviewer | Former general practitioner. Interviews form part of PhD research. |
| 8. | Interviewer characteristics | She has a background as a GP and an affinity for geriatric care. Working with an interviewer who is trained as a GP may have encouraged the participants to speak frankly and directly from their own professional perspectives. The second coder of the first phase has substantial experience in interview analysis but no medical background, which helped us avoid a ‘medical’ bias in our data interpretation. The second coder of the second phase is an expert on shared decision-making but has no background in practicing medicine. |
| Domain 2: Study design |  |  |
| Theoretical framework |  |  |
| 9. | Methodological orientation and theory | Thematic analysis (first phase) of all interview topics and framework analysis (second phase) of certain themes derived for the purpose of this study. |
| Participant selection |  |  |
| 10. | Sampling | A purposive snowball method aimed at recruiting professional experts. |
| 11. | Method of approach | Email. Some of the recruitment of GPs took place at a broader meeting of GPs specialised in geriatric care. |
| 12. | Sample size | 33 (18 CGs and 15 GPs) |
| 13. | Non-participation | The response rates of clinical geriatricians and general practitioners were 86% and 54%, respectively. Of the 21 CGs approached, one CG refused and two CGs did not respond to the first and reminder emails. A total of 28 GPs were approached. There were six non-responders, while three GPs responded positively but did not reply to the proposed dates. There were two drop-outs (the interview was cancelled and there was no response to the proposed rescheduled dates). Two GPs from the same practice chose one participant.  Lack of time was the main reason why some clinicians chose not to participate. |
| Setting |  |  |
| 14. | Setting of data collection | Five interviews were face-to-face, the others were held by telephone, as the medical practitioners’ busy schedules and varying locations required flexibility.  The face-to-face interviews were held at the interviewee’s office. |
| 15. | Presence of non-participants | No |
| 16. | Description of sample | See Table 2. Basic characteristics of participants |
| Data collection |  |  |
| 17. | Interview guide | Main topics and subtopics are provided in Additional file 2. A more detailed interview guide (in Dutch), including the introduction and closing of the interview, the specific questions on the topics and questions on basic characteristics, is available upon request. In one case, the interview guide was sent to the interviewee in advance of the interview. |
| 18. | Repeat interviews | No |
| 19. | Audio/visual recording | Audio recording of all interviews |
| 20. | Field notes | Yes |
| 21. | Duration | Approximately one hour. |
| 22. | Data saturation | Yes |
| 23. | Transcripts returned | Three interviewees wanted to receive the transcripts, which they were given. |
| Domain 3: Analysis and findings |  |  |
| Data analysis |  |  |
| 24. | Number of data coders | 2 |
| 25. | Description of the coding tree | The coding tree of the first phase of the analysis is available upon request. The categorisation matrix (second phase) is reported. |
| 26. | Derivation of themes | A thematic analysis was used for data analysis in the first phase to derive themes. In the first phase, we used open coding. The first five interviews were coded independently by two data coders (NV, MH). The initial codes were compared, discussed, grouped and categorised to determine a working analytical framework. The remaining interviews were coded by one researcher (MH) and checked by the other (NV). In weekly meetings, the researchers (NV and MH) compared, discussed and agreed on the coding of the transcripts, including the creation of additional codes and the further refinement of the analysis. In the second phase of the analysis, we further analysed the themes ‘SDM concept’ and ‘Links between the concepts of SDM and CGS’. For this purpose, we used a SDM categorisation matrix based on the essential, ideal and general elements of the integrative SDM model developed by Makoul et al., shown in Table 1 [18]. We added a new category to this SDM categorisation matrix: goals/goal setting. We charted the data in this categorisation matrix and findings were interpreted. |
| 27. | Software | Atlas-ti 7.1.15 |
| 28. | Participant checking | Two participants provided feedback upon request. |
| Reporting |  |  |
| 29. | Quotations presented | Yes |
| 30. | Data and findings consistent | Yes |
| 31. | Clarity of major themes | Not applicable |
| 32. | Clarity of minor themes | Not applicable |

*Note:* Consolidated Criteria for Reporting Qualitative Studies (COREQ): A 32-item checklist [41]
