# Supplementary material for: Goal setting is insufficiently recognised as an essential part of shared decision-making in the complex care of older patients: a framework analysis
Source: BMC Fam Pract. 2019 Jun 6;20:76. doi: 10.1186/s12875-019-0966-z (PMC6555756; doi:10.1186/s12875-019-0966-z)
Supplement: Supplementary file 2 — Table S2. Main topics and subtopics of the semi-structured interview guide. (DOCX 21 kb) [file 12875_2019_966_MOESM2_ESM.docx]

| **Table S2.** Main topics and subtopics of the semi-structured interview guide | | |
| --- | --- | --- |
| Main topics | | Subtopics |
|  |  | |
| Introduction of the interview | |  |
| Collaborative goal-setting between medical practitioners and patients | | - Definition of the concept - Experiences and process description - Types of goals - Barriers and facilitators |
|  | |  |
| Collaborative goal-setting within a collaborative framework of multiple medical practitioners | | - Experiences and expectations - Roles - Barriers and facilitators |
|  | |  |
| Shared decision-making between medical practitioners and patients | | - Definition of the concept - Experiences and expectations - Barriers and facilitators |
|  | |  |
| Shared decision-making within a collaborative framework  of multiple medical practitioners | | - Experiences and expectations - Roles - Barriers and facilitators |
|  | |  |
| Effective collaborative action between multiple medical practitioners | | - Definition of the concept - Experiences and expectations - Roles - Barriers and facilitators |
|  | |  |
| Relationships between the examined concepts of collaborative goal-setting, shared decision-making and effective collaborative action | | - Relationships between the concepts - Desirability of these processes - Possible actions to stimulate |
| Conclusion of the interview | | - Conclusion of the interview |
